# Self or Non-Self? It Is also a Matter of RNA Recognition and Editing by ADAR1

**DOI:** 10.3390/biology11040568

**Published:** 2022-04-08

**Authors:** Valentina Tassinari, Cristina Cerboni, Alessandra Soriani

**Affiliations:** Laboratory Affiliated to Istituto Pasteur Italia-Fondazione Cenci Bolognetti, Department of Molecular Medicine, Sapienza University of Rome, 00161 Rome, Italy; valentina.tassinari@uniroma1.it (V.T.); cristina.cerboni@uniroma1.it (C.C.)

**Keywords:** ADAR1, IFN, RNA editing, immune system

## Abstract

**Simple Summary:**

A fundamental feature of innate immune cells is to detect the presence of non-self, such as potentially harmful nucleic acids, by germline-encoded specialized receptors called pattern recognition receptors (PRRs). ADAR1 is one key enzyme avoiding aberrant type I interferon (IFN-I) production and immune cell activation by the conversion of adenosine to inosine (A-to-I) in double-stranded RNA (dsRNA) structures that arise in self mRNA containing specific repetitive elements. This review intends to give an up-to-date and detailed overview of the ADAR1-mediated ability to modulate the immune response in autoimmune diseases and cancer progression.

**Abstract:**

A-to-I editing is a post-transcriptional mechanism affecting coding and non-coding dsRNAs, catalyzed by the adenosine deaminases acting on the RNA (ADAR) family of enzymes. A-to-I modifications of endogenous dsRNA (mainly derived from *Alu* repetitive elements) prevent their recognition by cellular dsRNA sensors, thus avoiding the induction of antiviral signaling and uncontrolled IFN-I production. This process, mediated by ADAR1 activity, ensures the activation of an innate immune response against foreign (non-self) but not self nucleic acids. As a consequence, ADAR1 mutations or its de-regulated activity promote the development of autoimmune diseases and strongly impact cell growth, also leading to cancer. Moreover, the excessive inflammation promoted by *Adar1* ablation also impacts T and B cell maturation, as well as the development of dendritic cell subsets, revealing a new role of ADAR1 in the homeostasis of the immune system.

## 1. Introduction

The concept of discrimination between self and non-self has been associated for a long time with major histocompatibility complex (MHC) molecules and antigenic peptides recognized by the T cell receptor, and with the acquisition of central tolerance and avoidance of autoimmunity [1]. However, in the more recent past, it has become common to use the same terms for nucleic acids as well. In particular, and in relation to RNA, it is now well established that endogenous self RNA transcripts can form double-stranded RNAs (dsRNAs) resembling pathogen-derived dsRNAs and can be thus recognized as non-self by cytosolic RNA sensing receptors [2]. These, in turn, would activate type I interferon (IFN-I) signaling and production, resulting in a potentially dangerous autoimmune reaction. ADAR1 is one key enzyme avoiding such inappropriate activation in normal tissues, as it mediates dsRNA unwinding by deaminating adenosine and converting it to inosine (i.e., A-to-I conversion), making dsRNAs unable to be recognized by cellular receptors [3]. Indeed, the absence of ADAR1 in humans leads to Aicardi–Goutières syndrome (AGS), a severe interferonopathy characterized by high IFN-I production, IFN-stimulated gene (ISG) expression and autoimmunity-like symptoms [4,5,6,7]. In mice, cell-type-specific ablation of *Adar1* affects the normal maturation of T and B lymphocytes and dendritic cells (DCs), with characteristics of enhanced IFN-I/ISG signatures, resembling autoimmunity [8,9,10]. On the other hand, increased ADAR1 expression and/or activity can lead to cancer as it suppresses IFN-I production and anti-tumor immune responses [11,12,13]. In addition, since, upon A-to-I conversion, the cell interprets inosine as guanosine, RNA editing can result in non-synonymous codon changes in transcripts, and/or it can affect alternative splicing and miRNA targeting or maturation, giving rise to a very diverse transcriptome [14].

This review will provide an overview of these two opposing aspects of ADAR1 that, by modulating immune responses, affect autoimmunity and cancer.

## 2. RNA Recognition: Self versus Non-Self RNA and Activation of the IFN Pathway

**Figure 1:**
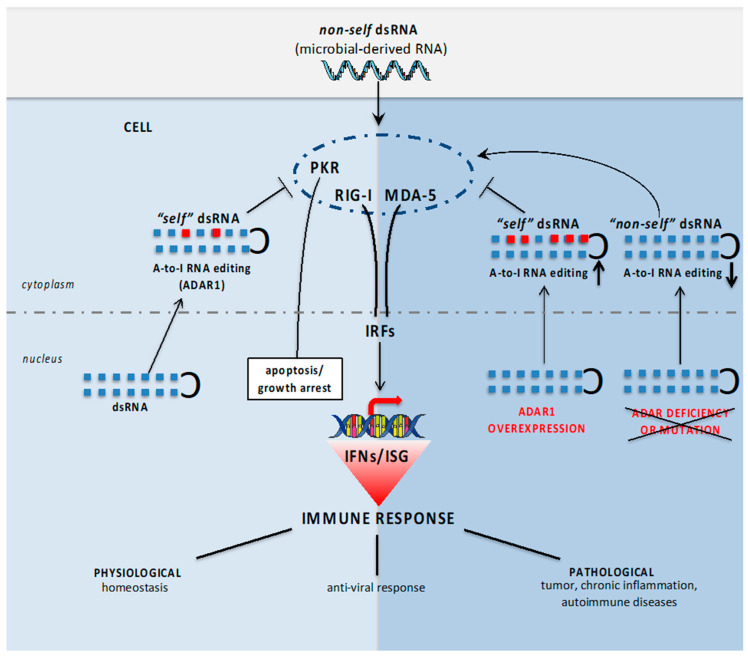
Effect of ADAR1-mediated A-to-I editing on endogenous and exogenous dsRNA. In the presence of ADAR1, endogenous dsRNA is edited (red square) and does not activate MDA5, RIG-I and PKR (homeostasis, normal tissues). In case of ADAR1 deficiency or mutation, endogenous dsRNA is not edited and recognized as non-self, as for exogenous microbial RNA, leading to IFN (IFN-I and -III) production, a strong ISG signature and uncontrolled immune response activation (chronic inflammation, autoimmune diseases). On the contrary, when ADAR1 is overexpressed, IFNs and their ISGs are dampened, limiting immune responses (tumor).

Innate immunity against pathogens is driven by specialized pattern recognition receptors (PRRs) that recognize structures unique to microbes (known as pathogen-associated molecular patterns, PAMPs) [15]. Viral dsRNAs may act as PAMPs and thus trigger the activation of specific PRRs, particularly the melanoma differentiation-associated protein 5 (MDA5) and the retinoic acid-inducible gene I (RIG-I) [15]. Upon dsRNA binding, these receptors interact with the mitochondrial antiviral signaling protein (MAVS) to form an RNA–protein complex, which triggers the downstream phosphorylation of the transcription factors IFN regulatory factor 3 and 7 (IRF3 and IRF7), thus promoting the expression of antiviral type I and III IFN (IFNα, IFNβ and IFNλ). The resulting secreted IFNs inhibit virus infection by inducing the expression of ISGs—including protein kinase R (PKR), 2′-5′-oligoadenylate synthetase 1-(OAS1), ADAR1 and ribonuclease (RNAse) L—in the original and in neighboring cells [16,17]. On the other hand, a large number of endogenous self dsRNAs are also naturally produced in eukaryotic cells under normal conditions. These transcripts, which originate from repetitive DNA, harbor inverted copies of the same repeat, and fold to create endogenous self dsRNAs that, similarly to viral dsRNAs, may stimulate cellular sensors to inadvertently activate IFN responses [2,18] (Figure 1).

Therefore, mechanisms regulating the balance among the immune sensing of foreign nucleic acids versus tolerance to the abundant self ones are of fundamental importance as they ensure antiviral defense and, at the same time, prevent immune disorders. In fact, uncontrolled IFN signaling has been associated with various pathological conditions, such as chronic inflammation, autoimmune diseases and cancer [19,20].

Recent studies have shown that the induction of cytoplasmic innate immunity and IFN production is regulated at the level of RNA by modifications such as methylation at the N6 position of adenosine (m^6^A) and A-to-I RNA editing. m^6^A is a co-transcriptional process catalyzed by proteins forming the m^6^A methyltransferase complex, where METTL3 is the catalytic subunit. Besides its role in regulating RNA stability, splicing, nuclear export and localization, recent discoveries support a role of m^6^A in regulating innate immunity by (i) preventing RIG-I sensing of viral or self-RNA and (ii) controlling the expression of specific genes that promote or inhibit innate immune responses [21]. Importantly, Winkler et al. demonstrated that the binding of the m^6^A “reader” YTHDF2 to methylated *IFN-β* RNA marks it for degradation, avoiding its prolonged expression and autoimmune dysfunctions [22].

Nevertheless, the main modification affecting RNA and ensuring immune homeostasis is A-to-I RNA editing, a post-transcriptional mechanism inversely correlated with m^6^A modification and catalyzed by ADAR enzymes, which will be further discussed below [3,23,24,25].

### 2.1. ADARs, A-to-I Editing and the Control of Innate Immune System Activation

A-to-I RNA editing refers to the deamination of specific A into I in dsRNA sequences. It is catalyzed by the ADAR family of enzymes, and it can strongly impact both the transcriptome and proteome diversity of eukaryotic cells [26,27]. The ADAR protein family is composed of ADAR1, ADAR2 and ADAR3. ADAR1 and ADAR2 are ubiquitously expressed, while the catalytically inactive ADAR3 is mainly found in the brain [17]. Two isoforms transcribed from alternative promoters are known for ADAR1: the shorter and constitutive ADAR1p110, and the N-terminally extended and IFN-inducible ADAR1p150 [28,29]. ADAR1p110 is mainly found in the nucleus, where it edits dsRNA before nuclear export [30], while, even if predominantly cytoplasmic, the ADAR1p150 isoform can shuttle between the nucleus and the cytoplasm, where it has been shown also to edit viral RNAs [28]. ADAR enzymes share a similar structure with a conserved N-terminal catalytic adenosine deaminase domain, and two or three dsRNA-binding domains (dsRBDs), by which they recognize and bind to both coding and non-coding RNAs (e.g., miRNA). Furthermore, both ADAR1 isoforms share the Z-DNA/RNA (Zα) binding domain, while a Zα domain is only expressed by ADAR1p150 [31]. 

Identification of edited sites depends on RNA sequencing data (RNA-seq). During translation, and upon RNA-seq, inosine is recognized as a guanosine (G) and A-to-I editing events are thus specifically identified as an A-to-G mismatch between the RNA-seq data aligned to the reference genome [32]. The functional consequences of ADAR activity are different. Novel protein variants arise from the so-called “recoding” editing, which consists of a non-synonymous A-to-G conversion in the protein coding region of the mRNA, thus changing the protein function or modifying splice site (generated or disrupted). In contrast, “non-coding” editing is produced when the conversion affects non-coding RNAs or non-coding parts of a specific mRNA [14]. 

In humans, a few “recoding” events have been identified and most of them are dependent on ADAR2 activity [14,33]. One such example is the recoding event affecting the *GRIA2* transcript, which encodes the main subunit of the glutamate receptor 2, and results in a glutamine-to-arginine change (or Q/R). This event is essential for life in mice as it results in an ionic channel that is impermeable to Ca^2+^ [34]. In fact, *Adar2^−/−^* mice normally die within 3 weeks of birth, but they are completely rescued when modified to constitutively express an arginine instead of a glutamine at the Q/R site [35,36]. 

In contrast, the majority of ADAR-edited sites detected occur in long dsRNA structures derived from a class of short interspersed elements (SINEs) called *Alu* elements, commonly found in introns and 3′-untranslated regions (UTR). As a consequence, in humans, most of the pre- or mature mRNAs form dsRNA structures and millions of ideal targets for ADAR enzymes’ activity are found within the cell [37,38,39,40,41]. ADAR1 is the enzyme that preferentially edits *Alu* elements, and, in 2014, Mannion et al. reported that ADAR1-dependent A-to-I modifications of cellular RNAs are a tolerance mechanism by which cells prevent the aberrant activation of innate immune sensors by host nucleic acids. Indeed, edited self dsRNAs have reduced integrity that prevents the binding to MDA5, avoiding aberrant IFN production [3]. Later, other laboratories further demonstrated that ADAR1 is a negative regulator of intracellular innate immunity responses, mainly by blocking the MDA5/MAVS axis. In particular, ADAR1 ensures that the amount of structured dsRNA is kept below a tolerance threshold for MDA5 sensing, while, in its absence, immunogenic dsRNAs exceed the tolerable number, leading to autoimmunity [3,23,24,42]. 

Concerning the fate of edited dsRNAs, further studies have been recently performed. Solomon et al. reported that A-to-I editing by ADAR1 may destabilize a small subset of transcripts characterized by more perfect dsRNA with A-uridine (U) base pairs, creating multiple I-U mismatches. These dsRNAs “destabilized when edited” may include *Alu* repeats within the 3′UTR of genes, which would be presumably recognized by dsRNA sensors if not edited by ADAR1. Interestingly, these *Alu* are mainly found within genes whose expression is upregulated during a type I IFN response, thus further supporting a role for ADAR1 in dampening innate immune responses. On the other hand, the favorite substrates for ADAR1 activity are “less perfect” dsRNA with A-C mismatches, where editing results in the stabilization of the structure [12,43].

Notably, another role of ADAR1 in regulating the antiviral response is by inhibiting PKR activity. Indeed, viral dsRNA also activates PKR, which, by phosphorylating eukaryotic initiation factor 2α (eIF2α), inhibits 5′-cap-dependent translation, blocking protein synthesis and preventing viral replication. In 2018, Chung et al. demonstrated that ADAR1 modulates the innate immune response not only preventing the unwanted MDA5 activation by self dsRNA, but also by inhibiting PKR activation during the IFN-I response, thus preventing translation shutdown and growth arrest [44] (Figure 1). 

### 2.2. Mutations in ADAR1

The role of ADAR1-mediated A-to-I modifications in the control of PRR activation is also clearly shown by murine model studies. *Adar1^−/−^, Adar1 p150*-specific knockout (KO) mice and *Adar1^E861A/E861A^* mice with a point mutation in the catalytic domain are all embryonically lethal, with overproduction of IFN-I and upregulation of ISGs [45,46,47]. These models thus demonstrate that ADAR1-dependent A-to-I editing is essential for early development in mice and in the control of IFN production [23]. Important findings were subsequently presented by different laboratories reporting how the concurrent deletion of the cytoplasmic sensors *Mda5* or *Mavs* completely rescued the embryonic lethality of *Adar1* and *Adar1p150* KO to live birth and allowed a normal lifespan in *Adar^E861A/E861A^* knock-in mice. Rescued mice also showed a reduction in the exacerbated IFN-I production [3,23,24,48]. Data from these animal models clearly pointed out that (i) RNA editing alters dsRNA structures to prevent MDA5 sensing of endogenous dsRNA; (ii) the sole p150 isoform is required to prevent MDA5 activation, supporting, as previously reported, the finding that *Alu* and evolutionarily related repeated elements located in the 3′UTR of mRNA are the predominant ligands for MDA5 signaling [42]. 

Of note, in humans, elevated IFN-I production and ISG expression in the absence of infection are found in patients with AGS, a severe, rare, autosomal recessive encephalopathy where ADAR1 is one out of the seven genes potentially mutated [4,5,6,7]. The mutations are mostly located in the catalytic domain; thus, it is reasonable to postulate that the pathogenesis of AGS is, in part, related to reduced A-to-I editing [3]. In addition, some patients show heterozygous point mutations affecting the Zα domain (i.e., N173S or P193A), together with mutations in the other allele affecting the deaminase domain or leading to loss of ADAR1 p150 expression. Since the Zα domain allows the p150 isoform to interact with nucleic acids adopting the unusual Z-conformation, patients with these mutations cannot interact with Z-RNA. The observation that the loss of Z binding prevents these patients from compensating for the absence of ADAR1 activity/expression caused by mutations in the other allele (in terms of elevated ISGs expression) suggests the important contribution of the Zα domain in the discrimination between self and non-self to prevent genetic diseases such as AGS [10,49,50,51,52].

## 3. ADAR1 in the Immune System

The capacity of ADAR1 to suppress aberrant ISG induction has been demonstrated to be crucial also in the development of thymic T cell maturation and in the maintenance of T cell homeostasis [53]. The thymus is a critical primary lymphoid organ, where T cells develop, mature and acquire self-tolerance to self-peptides displayed by MHC molecules. In a recent study, Nakahama et al. reported that ADAR1 is abundant in the mouse thymus and its expression is upregulated during T cell maturation, especially at the CD4+CD8− single positive stage, together with multiple ISGs [54]. The specific deletion of *Adar1* in murine CD4+ T cells reduced both populations of mature CD4+ and CD8+ single positive T cells, resulting in impaired T cell maturation. The authors also demonstrated that unedited dsRNAs trigger MDA5 activation, which leads to excessive ISG expression and defective TCR signaling. This was thought to be the cause of the defect in T cell maturation, since ISGs and IFN-I inhibit TCR signal transduction and T cell proliferation. Strikingly, the same *Adar1* KO mice showed loss of self-tolerance and induction of autoimmunity, leading to spontaneous colitis with the accumulation of T cells in the lamina propria. Thus, this study suggests that ADAR1 is required for establishing central tolerance during thymocyte development by preventing the MDA5 sensing of endogenous dsRNA as non-self. However, it also raises important considerations regarding ADAR1. First, ADAR1 has distinct functions in different cell types. In general, a lack of ADAR1 leads to apoptosis and cell death [23,44], while, in thymocytes, it affects cell maturation. Second, it may play an active role in maintaining moderate (or low) ISG expression in developing T cells, which may be crucial for thymic T cell maturation and the acquisition of self-tolerance. As a consequence, the work by Nakahama et al. raises the possibility that autoreactive T cells could be a contributing factor not only in AGS patients with defective *Adar1*, but also in autoinflammatory disorders, such as lupus erythematosus (SLE), where enhanced ISG expression is one of the traits [55]. Excessive ISG expression in T cells could thus impair T cell maturation/selection, causing or exacerbating a breakdown in thymic self-tolerance in SLE. In line with these considerations, primary CD4+ T lymphocytes isolated from ADAR1-deficient AGS patients showed upregulated expression of ISGs and were more resistant to HIV-1 infection [7,56].

In relation to the acquisition of central tolerance in the thymus, it is interesting to mention that medullary thymic epithelial cells (mTECs) display significantly higher rates of A-to-I RNA editing (including recoding), as well as of other transcriptional and post-transcriptional processes, compared to other cells and tissues in the body [57]. mTECs are the main players in the educational program of T cells in the thymus as they have a unique capacity to express and present a large fraction of body antigens to T cells, leading them to discriminate between self and non-self molecules [58]. Since recoded proteins through RNA editing can be processed and loaded into MHC molecules as self-peptides, they could be then recognized as neo-self-antigens in the periphery to trigger autoimmune responses. Therefore, the significantly increased levels of RNA processing and editing in mTECs further expand the diversity of their self-antigen repertoire and are probably important for the acquisition of self-tolerance against edited-self, the elimination of autoreactive T cells and the prevention of autoimmunity [57].

ADAR1 was shown to be crucial also for B cells [9,24]. Normal B cell development in the mouse bone marrow was affected by a B-cell-specific CD19-Cre-mediated *Adar1* ablation. This caused a significative defect in the final stages of B cell maturation, with an almost complete absence of newly formed immature and CD23+ mature B cells in the bone marrow, as well as reduced peripheral blood and splenic B cells [9]. Of note, in this experimental model, the ablation of *Adar1* was also accompanied by enhanced ISG expression and apoptosis.

Analysis of the role of ADAR1 in the context of other leukocyte populations has revealed its importance in macrophages and DCs. In primary macrophages, *Adar1* knockdown significantly enhanced the production of IFN-I and of pro-inflammatory cytokines and chemokines, which in turn had antiviral paracrine activity, as they inhibited HIV-1 replication in bystander cells [59]. Conditional in vivo ablation of *Adar1* in murine CD11c+ cells had systemic effects on the development of discrete DC subsets, particularly lung CD8+/CD103+ DCs as well as alveolar macrophages, whereas apoptosis, which is generally observed in multiple tissues of *Adar1* KO mice, was not induced [8]. Analysis of NGS-derived RNA sequences from ADAR1-deficient alveolar macrophages showed a dramatic loss of A-to-I RNA editing both in coding and in long non-coding RNAs, further supporting the cell-type-specific effects of RNA editing by ADAR1 [8].

Another study analyzing editing levels and edited sites in human blood using RNAseq data from 459 healthy individuals has revealed more than 2000 sites consistently edited, with ADAR1 emerging as the major contributor to editing processes in blood [60].

Altogether, these data clearly demonstrate that the ADAR1-mediated suppression of IFN-I signaling is important not only for innate immune cells, but also for the development and homeostasis of adaptive immune cells (Table 1, Figure 1).

## 4. ADAR1 and Its Role in Cancer

Although both ADAR1 and ADAR2 family members have been associated with neoplastic transformation, ADAR1 is commonly overexpressed in cancer cells [61,62,63]. ADAR1’s contribution to cancer is mediated by different mechanisms. Among them, the most debated are (i) A-to-I editing of certain mRNAs, which creates proteins with pro-tumoral functions [64,65,66,67]; (ii) editing of non-coding parts of pro-tumoral mRNAs, which could prevent their degradation by miRNAs [68]; (iii) editing of non-coding RNAs, which could affect cancer-related miRNA biogenesis [69,70]; (iv) editing-independent mechanisms [63]; (v) editing of immunogenic dsRNAs, which could prevent inflammation, enhancing tumor malignancy. In relation to the last aspect, cancer cells could take advantage of ADAR1 overexpression to avoid IFN production and ISG gene expression, thus establishing a mechanism to escape recognition/activation of the immune system. Accordingly, recent studies showed that cancer-derived cell lines are dependent on ADAR1 for their survival, and this phenotype depends on the ability of this enzyme to modulate the IFN pathway [11,13]. As reported by Liu et al. and by Gannon et al., a high ISG gene expression signature is predictive for the ADAR1 dependence of several cancer cell lines, and this vulnerability seems to be mediated by the exacerbated activation of different dsRNA sensors, such as PKR and MDA5, in the absence of this enzyme. MDA5 activation, against an ADAR1-null background, increases IFN-I production, promoting inflammation and immune infiltration, while PKR activation leads to translation inhibition and growth arrest (Figure 1). Notably, in ADAR1-silenced cancer cells, cell viability was restored only after concomitant PKR deletion [11,12,13,44]. Because cancer cells can produce IFN-I per se, the vulnerability to ADAR1 loss might be different between cancerous and normal tissues [13]. Indeed, while deleterious for normal cells, the persistent activation of dsRNA sensor pathways reduces cancer cell viability, thus representing a promising therapeutic anti-cancer strategy. This is also demonstrated using anticancer epigenetic inhibitors able to induce the transcription of repeated sequences that form dsRNAs [71,72]. Additionally, *Adar1* deletion in mice reduces tumor growth and increases tumor inflammation, promoting an increase in CD8^+^ T cells infiltrating tumors, and it sensitizes mice to anti-PD-1 antibody [12]. 

Altogether, these findings point at ADAR1 as a key molecule in the intracellular pathways regulating IFN production, ISG expression and thus both innate and adaptive immune responses. 

## 5. Conclusions

Here, we carried out a comprehensive analysis of recent findings that indicate ADAR1-dependent A-to-I editing as a mechanism of immune repression to prevent the activation of antiviral pathways in normal conditions. Indeed, the main biological function of ADAR1 seems to be the ability to alter endogenous dsRNA structures by A-to-I editing, avoiding their recognition by dsRNA cellular sensors (e.g., MDA5). This mechanism ensures cellular homeostasis, keeping in check the expression of IFNs, ISGs and pro-inflammatory cytokines. Furthermore, besides its role in the control of innate immune system activity, ADAR1 is also important for the development of adaptive immune cells. 

It is not surprising that ADAR1 deficiencies promote autoimmune diseases with excessive IFN signaling, as in AGS, for which no targeted therapies are currently available. Thus, progresses in understanding AGS pathogenesis may lead to attempts at more specific therapies, through either a limitation in the production of self nucleic acids (or an increase in their degradation), or by blocking downstream intracellular pathways leading to inflammation. Antibodies against IFNs or their receptors, and/or small molecules antagonizing the production and signaling of specific self-derived nucleic acid species, including dsRNAs, will hopefully become available in the short to medium term, with important clinical gains for this devastating disease. 

On the opposite side, rises in ADAR1 activity can contribute to cancer progression, by repressing inflammation and avoiding immune cell activity. Indeed, ADAR1 inhibition results in reduced cell growth, reactivation of the IFN pathway and the expression of hundreds of ISGs, likely reshaping immune responses and enhancing the ability of immune cells to reach the tumor site. 

The findings that we have discussed herein contribute to the growing literature supporting ADAR1 as a promising new immune-oncology target and paving the way to finding novel cancer therapeutics targeting this enzyme. ADAR1 inhibitors, able to reduce its editing activity or its expression, may potentially represent a therapeutic perspective for several cancers. At the same time, cancer treatments targeting ADAR1 should be evaluated for their ability to synergize with currently available therapies, such as epigenetic inhibitors.

## Figures and Tables

**Table 1:** Effect of ADAR1 mutations on cells of the immune system.

Cell Type/Organ	Mouse/ Human	Mutation/Gene Deficiency	Functional Effects	Reference
Single positive thymocytes	Mouse	*Adar1* deletion in CD4+ T cells	Impaired T cell maturation, excessive ISG expression, defective TCR signaling, induction of autoimmunity (spontaneous colitis)	[54]
Primary CD4+ T lymphocytes	Human	*Adar1*-deficient AGS patients	Upregulated ISG expression, resistance to HIV-1 infection	[7,56]
mTECs	Mouse	None	Demonstration of high rates of editing events, acquisition of self-tolerance against edited-*self*, prevention of autoimmunity	[57]
B lymphocytes	Mouse	*Adar1* deletion in CD19+ B cells	Impaired B cell maturation, reduced numbers of peripheral blood and splenic B cells, enhanced excessive ISG expression and apoptosis	[9]
Primary macrophages	Human	siRNA ADAR1 silencing	Enhanced production of IFN-I, chemokines and cytokines, inhibition of HIV-1 infection in bystander cells	[59]
DCs	Mouse	*Adar1* deletion in CD11c+ cells	Peripheral systemic loss of CD103+/CD8+ DCs, reduced number and dysfunction of AM, loss of RNA editing in AM	[8]

Abbreviations: mTECs, medullary thymic epithelial cells; DCs, dendritic cells; AM, alveolar macrophages.

## Data Availability

Not applicable.
